# The Co-Evolution of Network Structure and PrEP Adoption among a Large Cohort of PrEP Peer Leaders: Implications for Intervention Evaluation and Community Capacity-Building

**DOI:** 10.3390/ijerph18116051

**Published:** 2021-06-04

**Authors:** Lindsay E. Young, John A. Schneider

**Affiliations:** 1 Annenberg School for Communication and Journalism, University of Southern California, 3502 Watt Way, Los Angeles, CA 90089, USA; 2 Chicago Center for HIV Elimination, University of Chicago, Chicago, IL 60637, USA; jschnei1@medicine.bsd.uchicago.edu; 3 Departments of Medicine and Public Health Sciences, University of Chicago, Chicago, IL 60637, USA; 4 Crown School of Social Work Practice and Policy, University of Chicago, Chicago, IL 60637, USA

**Keywords:** social network interventions, peer leaders, HIV prevention, capacity-building, stochastic actor-based models

## Abstract

**Background:** Peer leader interventions are effective strategies for promoting prevention behaviors in communities at risk for HIV, yet little is known about their effects on the social and behavioral dynamics of peer leaders themselves. **Methods:** Using data from PrEP Chicago, an RCT PrEP for prevention intervention for young Black MSM (YBMSM), we apply stochastic actor-based models to longitudinally model the impact of study participation on the online friendship and PrEP adoption dynamics among a network of peer leaders (*n* = 174) and a network of control group counterparts (*n* = 166). **Results:** Peer leaders assigned to the same leadership training workshop were more likely to form new Facebook friendships with one another, whereas control participants assigned to the same attention control workshop were no more or less likely to form new friendships. Further, peer leaders with greater PrEP intentions and those living with HIV were more active in forming new friendships with other peer leaders, effects not found in the control network. PrEP adoption was not influenced by network dynamics in either group. **Conclusions:** The implications of these findings are discussed through the lens of community-capacity building and the role that peer leader interventions and the networks they engage can impact public health.

## 1. Introduction

Despite the clear efficacy of Pre-Exposure Prophylaxis (PrEP) in preventing HIV transmission, meaningful uptake in populations experiencing high HIV incidence, most notably young Black gay, bisexual, same gender loving and other men who have sex with men (hereafter YBMSM), has yet to occur [1,2]. In a population-based sample of YBMSM in 2014 for example, only 41% had ever heard of PrEP and 4% ever used it [3], despite this same population experiencing some of the highest rates of HIV in the United States [4]. Likewise, in PrEP demonstration and implementation projects in Washington, DC [5] and New York City, NY, USA [6], less than 15% of PrEP clients identified as Black, while only 31% of clients identified as Black in the CDC’s Sustainable Health Center Implementation PrEP Pilot (SHIPP) [7]. 

Although individual factors like low awareness, misperceptions about suitability, and concerns about side effects are common first-order barriers to PrEP uptake [8,9,10], deeper and more complicated social obstacles are also increasingly observed. For example, stigma associated with being perceived as HIV positive or sexually promiscuous have been linked to YBMSMs’ reluctance to consider PrEP [11]. Additionally, as intersectional racial and sexual minority identities [12], YBMSM must cope with homophobic and racist discrimination in multiple contexts, including clinical spaces, which can complicate their willingness to engage with traditional sources of HIV prevention messaging such as primary and sexual health care providers and public health researchers [13,14]. Given these challenges, innovative implementation strategies are clearly needed that can reach greater portions of most impacted communities like YBMSM and provide alternative paths to prevention services that are perceived by YBMSM as more accepting and less stigmatizing.

Decades of social diffusion research underscores the role that personal relationships, and the trust they engender, play in the adoption and spread of novel ideas and behaviors in a population [15]. More recently, social network interventions—intentional efforts to leverage network structure and peer influence processes to accelerate the diffusion process—have been advanced from a public health framework [16]. Perhaps the most intuitive network intervention is the peer leader intervention, where members of the prioritized population (i.e., peers) are positioned in the role of health educators who disseminate information about a health innovation through their personal networks [16,17,18,19]. 

Applied to the challenge of advancing PrEP, peer leader interventions offer the opportunity to reach larger portions of communities at risk for HIV seroconversion by treating social networks as opposed to individuals in isolation [20] while also privileging endogenous, community-based systems of communication and influence over institutionalized ones. Indeed, efforts to leverage peer leaders to promote PrEP awareness and early linkage among Black MSM, although few in numbers, show promise in this regard [21,22,23]. 

Unsurprisingly, the impact of peer leader interventions tends to be measured on the basis of observed changes in the health behaviors of network associates with whom peer leaders are trained to interact, not on the basis of change in the behaviors of the peer leaders themselves. This is because peer leaders are a network intervention’s active ingredient, not the focus of change. Although cogent arguments have been made that peer leader interventions have valuable secondary effects on the attitudes and behaviors of peer leaders themselves [24], rarely have these effects been rigorously evaluated (for exceptions see [25,26,27]). As a consequence, we know little about the way in which peer leader interventions impact the network and health behavior dynamics among the peer leaders themselves.

To understand the significance of learning how the evolving social and behavioral dynamics of peer leaders are impacted by their involvement in an intervention, it is vital to see peer leaders, collectively, as an investment in community capacity. Theories of community development underscore the importance of building capacity in communities facing adversity, meaning that community members’ abilities to become active agents (rather than objects) of change must be nurtured [28]. Two fundamental components of capacity building are: (1) investment in the development of local leaders who are empowered to help the community make good decisions, and (2) nurturing the formation of social networks that facilitate flows of information and support [28]. Endogenous leaders and the networks they form are, therefore, a public good, embedded with knowledge, experience, and social capital from which the community as a whole can benefit [29]. From this perspective, a peer leader intervention is more than just a means to diffuse information about an innovation like PrEP through the networks of YBMSM: it is also a means to strengthen the capacity and resilience of a marginalized community through its activation and nurturing of a network of young community health leaders [30,31,32]. The degree to which the intervention nurtures this system of human and social capital, however, depends on the collective capacity that the peer leaders generate among themselves through their own network-building [33,34,35] and their own buy-in with regard to the behavior they are being asked to promote (i.e., PrEP adoption). 

The objective of this study, then, is to ascertain whether the training and support provided by a PrEP peer leader intervention is indeed an engine of social and behavioral change among YBMSM peer leaders or whether these dynamics are more attributable to factors outside the scope of the intervention, for example individual characteristics or structural features of their naturally-evolving organic networks. To these ends, we draw on a novel dataset of social network and behavioral data collected from a large cohort of peer leaders in a community-based PrEP for prevention intervention for YBMSM living in Chicago, IL, USA. Using stochastic actor-based models (SABMs) [36,37,38], we longitudinally model the intervention’s impact on both friendship formation and PrEP adoption among peer leaders during the first year of their study enrollment. Further, given that the social and behavioral dynamics of peer leaders are likely to be interdependent, these models also allow us to simultaneously test the effects of behavior on friendship selection (i.e., when PrEP adoption informs friendship formation among peer leaders) and the effects of friendship on rates of behavior change (i.e., when friendships influence PrEP adoption). We anticipate that the results of our analysis will help identify where improvements to peer leader training and engagement may be needed to enhance and strengthen their capacity as a cohort of community health leaders. 

## 2. Materials and Methods

### 2.1. Study Site and Population

Data for this study were collected from March 2016–March 2018 as part of a randomized controlled trial peer leadership intervention among 423 YBMSM living primarily on the south and west sides of Chicago. Participants were considered eligible if they met the following criteria: (1) 18–35 years of age, (2) identified as Black or African American, (3) assigned male sex at birth, (4) had sex with a man in the past 12 months, and, because the intervention emphasized social media as a communication tool, (5) had an active Facebook profile. All data collection implicated in this study received ethics approval from the University of Chicago School of Medicine, Biological Sciences Division and from NORC at the University of Chicago.

Participants were recruited using respondent-driven sampling (RDS), a procedure well suited for identifying members of “hard-to-reach” populations like MSM [3]. A variant of snowball sampling [39,40], RDS draws on peer referral chains, beginning with a set of initial “seeds” that meet study eligibility. Because seeds should have large social networks (i.e., are popular) and have ties to a diverse array of people belonging to different subpopulations [40,41,42], we selected YBMSM seeds based on their central or boundary spanning positions (i.e., structural signatures of popularity and diversity, respectively) within a previously derived Facebook friendship network among the focal population [43]. Once a seed was enrolled and completed their baseline assessment, they were instructed to recruit up to six peers (or “sprouts”) who also met the eligibility criteria. Following enrollment, sprouts were also instructed to recruit peers, and the process continued until the recruitment target was reached. Participants received a $20 cash incentive for each peer whom they successfully referred into the study.

### 2.2. Study Design and Data Collection

The study design and data collection approach have been previously published in Young and Schumm [44]. To summarize, participants were assigned randomly to one of two treatment sequences: (1) receives the peer leader training in year 1 of the study (Year 1 intervention arm) or (2) receives peer leader training in Year 2 (Year 1 attention control arm). Here, we focus on Year 1 of the study, as the availability of an intervention network and control network allows us to compare the evolving network and behavioral dynamics in each group and ascertain whether the peer leader training itself (i.e., the treatment) impacts those dynamics. Once participants were randomized, they were scheduled for a baseline visit. All participants provided written consent during that baseline visit.

The peer leader training adapted the peer educational and mentoring program developed as part of the HIV Prevention Trials Network [45,46] and was conducted in small groups (6–10 people) in a single half-day workshop. The training curriculum was designed to develop an individual’s PrEP knowledge and their communication skills for engaging network associates in PrEP-related conversations. Participants not assigned to the peer leader training in Year 1 were assigned to a attention control condition that reproduced the nonspecific procedures used to engage with intervention participants (e.g., small group, half-day workshops led by study staff) without including its specific content [47] (see Young, Schumm [44] for more details). 

Data used in this study were collected at Baseline and 12-months. Collection modalities included: (1) a computer-assisted self-administered survey capturing information about PrEP knowledge and attitudes, sexual health behaviors, psychographics, and demographics; (2) biomedical HIV and STI testing; and, to evaluate the relationship between social connectivity and intervention outcomes, (3) a manual download of participant’s Facebook friendship data. A waiver of consent from the IRB for third party (non-participant) network members was obtained given the minimal risk to these individuals. Data protections to secure third party identities (e.g., hashing, numeric de-identification) were also established [20].

### 2.3. Measures

#### 2.3.1. Facebook Friendship Networks

With the Facebook friendship lists we acquired from consenting participants at baseline and 12-months, we constructed two sets of unweighted undirected edge lists—one set that represented Facebook friendship ties among intervention participants at each time point and another set representing Facebook friendship ties among control participants at each time point. In both sets of edge-lists, all ties to third-parties (i.e., non-participants) were excluded. 

#### 2.3.2. PrEP Adoption

PrEP use was measured on the basis of a participant’s self-report of being on PrEP at the time of the baseline and 12-month assessment, respectively. From these self-reports, we created a binary PrEP use variable (1 = currently taking PrEP; 0 = not currently taking PrEP). 

#### 2.3.3. Study Participation Effect

To account for how study participation influences the network and behavioral dynamics in each group, we include a dyadic measure of being in the same training cohort. Specifically, this represents having been assigned to the same peer leader training cohort (for participants assigned to the intervention arm) or the same risk assessment cohort (for participants assigned to the attention control arm). We interpret this particular dyadic covariate in each model as an effect of study participation on the ongoing social dynamics among study participants.

#### 2.3.4. Actor Covariate Attributes

Three actor attributes believed to be associated with PrEP adoption and/or the formation of Facebook friendships are also included in our analysis. First, we account for a participant’s age (measured at baseline), which has been shown to be related to Facebook connectivity in previous work [48] and that has also been linked to willingness to adopt PrEP [49]. Second, we include a measure of HIV status (1 = HIV positive; 0 = HIV negative) as this, too, has been linked to increased Facebook connectivity among YBMSM [48] and is an explicit eligibility criteria for PrEP adoption. HIV status was measured using biomedical lab testing (i.e., blood tests) or self-reports if lab tests were not available. Third, to account for the theorized relationship between behavioral intentions and behavioral adoption [50,51], we account for PrEP intentions [52] measured as the perceived likelihood that a participant would take PrEP in the next six months (1 = probably/definitely would not take PrEP; 2 = might take PrEP; 3 = probably/definitely would take PrEP).

#### 2.3.5. Dyadic Covariate Attributes

To account for expected relationships between offline social relationships and Facebook friendship dynamics, we also examine the effect of an offline physical-world dyadic attribute representing being tied to another participant through a referral connection. Although referrals are directed relationships, we include them here as non-directed ties to represent whether or not two actors have a physical world connection outside the study. 

### 2.4. Data Analysis

#### 2.4.1. Analytic Sample

The analytic sample is a subset (*n* = 340) of the study participants derived from who among the 423 enrolled participants consented to the Facebook data collection at both their baseline and 12-month assessments. Specifically, of the 423 YBMSM study participants at baseline, 346 were retained at 12-months. Of the 346 participants who were retained at 12-months, six either did not consent to the data collection or experienced technical difficulties when downloading their data at the time of data collection. This yielded a total of 340 participants for whom we have Facebook friendship data at both waves. The analytic dataset was then sub-divided into two sub-samples, one comprised of participants assigned to the intervention arm (*n* = 174) and the other comprised of participants in the control arm (*n* = 166). No significant differences were found between those who were retained and consented to the Facebook data collection (*n* = 340) and those who were not retained or did not consent (*n* = 83). 

#### 2.4.2. Actor-Based Models for Diffusion of Innovations in Dynamic Networks

To model interdependencies between changes in Facebook friendships among the YBMSM study participants and the rate of PrEP adoption among members of each sub-sample, we applied an extension of stochastic actor-based models (SABMs) [37,38] called the actor-based model for diffusion of innovations in dynamic networks [53]. All analyses were implemented using ‘RSiena’ (Simulation Investigation for Empirical Network Analysis) version 1.2-23 for the statistical system R version 4.0.2 [54]. 

A description of the foundational premise and underlying logic of these models have been published previously by the first author [48] and elaborated extensively by Snijders and Van de Bunt [36] and Greenan [53]. The foundational premise of these models is that an innovation (e.g., PrEP adoption) is not only dependent on the social network to which an individual belongs, but also on the changes in that social network [53]. As such, they model the co-evolution of social networks and behaviors. A more detailed explanation of the logic behind these models is available in Appendix A.

#### 2.4.3. Model Specification

Given that intervention participants were intentionally motivated to think and talk about PrEP and to engage with one another as a cohort of peer leaders, while control participants were not, we modeled the co-evolving network and behavioral dynamics of each subsample independently from one another, using the same model specifications. This allowed us to compare model results across treatment and control conditions to see how study enrollment differentially impacted each group and to better understand potential mechanisms of capacity building among peer leaders. The actor-based model for the treatment and control samples includes two sub-models that are estimated simultaneously: a network dynamics sub-model to predict changes to network members’ Facebook friendships, and an adoption process sub-model to predict the rate of PrEP adoption. 

#### 2.4.4. Network Dynamics

Facebook friendship ties were operationalized as symmetric (or non-directed) connections and modeled with the assumption that ties are unilaterally initiated and reciprocally confirmed, while confirmation is not required for tie dissolution [55]. These assumptions correspond to how friendships are formed on Facebook: friendship ties are formed when one user initiates a friendship request to another user and the recipient of that request confirms the request, while “de-friending” can be done unilaterally.

Guided by these assumptions, the network dynamics sub-model includes a rate function, capturing the speed of change in the Facebook network, and a set of evaluation effects that represent the mechanisms by which the dependent behavior (PrEP adoption), study participation (i.e., being a part of the same training cohort), other actor and dyadic covariates, and the network itself govern the formation of Facebook ties (see Table 1). Two evaluation effects tested the impact of the behavioral dependent variable (PrEP adoption) and each constant actor covariate (age, HIV status, and PrEP intentions) on changes to Facebook friendship ties: (1) the effect of either the behavior or covariate attribute on an actor’s propensity to form Facebook friendships (egoPlusAltX), and (2) the effect of assortativity on the behavior or covariate attribute, where actors are more likely to form friendships with other actors who share the same behavior and/or attribute (sameX). Additionally, we included a constant dyadic covariate term to represent the effect of a study-specific relationship between participants assigned to the same intervention or control training group. We also controlled for a dyadic covariate that represents the effect of a study referral relationship. 

Finally, we also controlled for several structural effects that represent the way in which Facebook friendships are formed in response to the presence or absence of other ties in the network. Specifically, we included: (1) a required degree effect that models the overall tendency for actors to form Facebook friendships (density), (2) an effect that represents the tendency to have network closure in Facebook friendships (geometrical-weighted edgewise shared partnerships (gwesp)), (3) a term representing the preference to form Facebook friendships with highly connected network members (i.e., actors with high Facebook degree) (degPlus), (4) an effect representing assortativity on Facebook degree (degree assortativity), and (5) an effect that models the tendency for network isolates to remain isolated (outIso). Structural parameters were chosen on the basis of theoretical considerations and the results of overall goodness of fit tests.

#### 2.4.5. Adoption Process

As demonstrated by Greenan [53], we model the PrEP adoption process as a proportional hazards model [56], meaning that we model at any given point in time the risk of a single actor adopting PrEP for the first time, conditional on the current state of the dynamic network. We consider three types of adoption effects in our adoption process sub-model (see Table 1). Total Exposure (totExposure) captures social influence conveyed through overt exposure and is measured by the total number of network contacts that are PrEP users [57]. Infection by degree (infectDeg) is a measure of how influential an actor’s PrEP use is on the rest of the system [58], where influence is operationalized in terms of their popularity on Facebook (i.e., Facebook friendship degree) [53]. Finally, we also consider how intrinsic characteristics affect an actor’s propensity to adopt PrEP, irrespective of the PrEP use of other network members, by considering the effects of an actor’s HIV status and their PrEP intentions.

## 3. Results

### 3.1. Descriptives

A summary of descriptive statistics for the behavior (PrEP adoption) and each constant attribute covariate for the intervention and control arm sub-samples is presented in Table 2. Results of tests of difference (not shown) revealed no significant differences between the intervention and control participants on these attributes.

Structural characteristics of each Facebook friendship network at baseline and 12-months and their dynamic features are summarized in Table 3. First, in both the intervention and control networks, an increase in Facebook connectivity among study participants during the first 12 months of the study is evident. Study participants in both arms of the study gained on average four friendships with other study participants between baseline and 12-month observations, which corresponded to a 0.02 increase in network density in both arms. Second, there were also slight increases in friendship closure among study participants in each study arm, made apparent by changes in transitivity: transitivity increased from 27% to 30% in the intervention arm and 25% to 31% in the control arm. Finally, although the global centralization of the friendship network is relatively low at baseline (16% concentration in the intervention arm and 18% concentration in the control arm), there was a 7% and 5% increase in friendship concentration around “hubs” in the intervention and control arm networks, respectively. 

**Figure 1:**
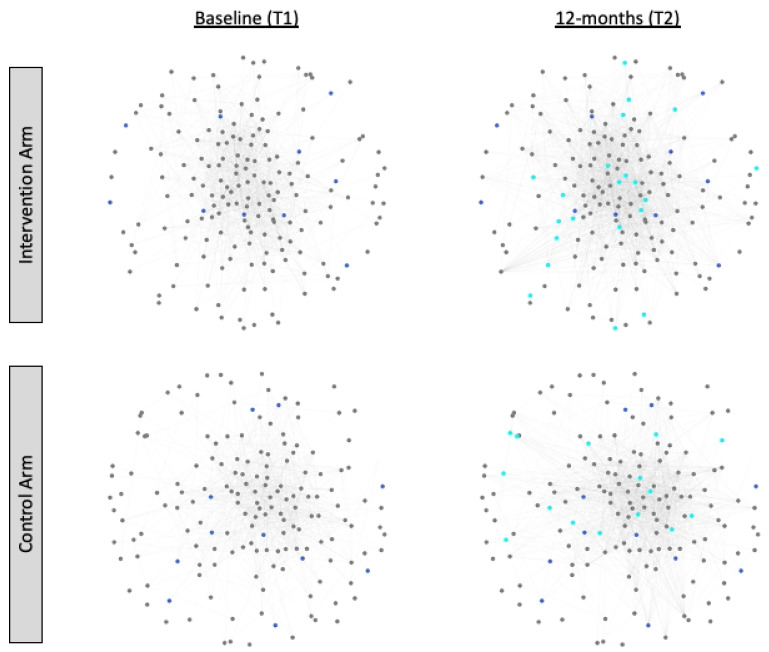
The Facebook friendship networks among intervention (*n* = 174) and control arm (*n* = 166) participants at baseline (T1) and 12-months (T2), with information about PrEP adoption status. Each circle (node) represents one study participant in either the intervention or control arm sub-samples. Circles are colored by their PrEP adoption status at each wave: grey denotes a study participant who was not a PrEP adopter, dark blue denotes a study participant who reported being on PrEP at baseline (T1), and aqua blue denotes a study participant who adopted PrEP at 12-months (T2). Visualizations were created in Python.

When we examine each network over time at a more granular tie-level, the network changes become more obvious. Between Waves 1 and 2, intervention participants added 555 new ties to their Facebook friendship network and control participants added 533 new ties to their Facebook friendship network. This equated to about 3.2 new friendships per person in each network. At the same time, 198 (17%) of the 1140 baseline friendships among intervention arm participants were dissolved, while 200 (25%) of the 813 baseline friendships among control arm participants were dissolved. In total, 942 (83%) and 613 (75%) of the baseline Facebook friendships observed among intervention and control arm participants, respectively, were maintained. Figure 1 shows the Facebook friendship networks among intervention and control arm participants at baseline and 12-months, with actor nodes colored by their PrEP adoption status at each wave.

### 3.2. Actor-Based Models for the Diffusion of PrEP in Dynamic Facebook Networks

#### 3.2.1. Model 1: Intervention Arm

In the intervention arm (Table 4, Model 1), the network dynamics sub-model reveals that the intervention arm participants were more likely to form and maintain Facebook friendships with one another if they were also co-members of the same peer leader training cohort (*b* = 0.92, *p* < 0.001). This lends support to the idea that the group training context can be an important ingredient in increasing the collective capacity of candidate peer leaders.

Although we observed an 8.5% increase in PrEP adoption among intervention participants between baseline and 12-months, results show that changes in PrEP adoption had no effect on the formation of Facebook friendships among intervention participants. Rather, actor covariates like age, HIV status, and PrEP intentions were more important mechanisms of network change. Specifically, intervention participants who were living with HIV (*b* = 0.19, *p* < 0.01) and who had greater intentions to adopt PrEP (*b* = 0.13, *p* < 0.01) were more active in forming friendships with other network members irrespective of the HIV status and PrEP intentions of those members. Intervention participants were also more likely to form friendships on the basis of age (*b* = 1.30, *p* < 0.001) and HIV status (*b* = 0.18, *p* < 0.05) similarities. Having an existing offline relationship in the form of a study referral tie (*b* = 0.67, *p* < 0.10) also influenced the formation of new Facebook friendships, although the significance of that effect was marginal.

Finally, the formation of Facebook friendships among intervention participants was also governed by the structure of the network itself. The formation of friendship ties was not done arbitrarily (negative degree term), but they were more likely to form friendships with the friends of their friends, ensuring network closure (positive gwesp term (*b* = 0.26, *p* < 0.05)), and more likely to form friendships on the basis of mutual popularity (positive outInAss term (*b* = 0.06, *p* < 0.01)).

The PrEP adoption process sub-model featured in model 1 shows that the rate of PrEP adoption among intervention participants was not influenced by their Facebook friendships; neither their connections to friends who were PrEP adopters (totExposure term (*b* = 0.13, *p* = n.s.)) nor their connections to influential PrEP adopters (*infectDeg* term (*b* = −0.01, *p* = n.s.)) impacted their PrEP adoption. Unsurprisingly, the rate of PrEP adoption was significantly predicted by the intrinsic effect of being HIV positive (*b* = −1.34, *p* < 0.05), which we included as a control variable. PrEP adoption was not, however, influenced by participants’ PrEP adoption intentions (*b* = 0.51, *p* = n.s.).

#### 3.2.2. Model 2: Control Arm

In the control arm (Table 4, Model 2), the network dynamics sub-model shows that being a part of the same training group had no effect on the formation and maintenance of Facebook friendships among control arm participants (*b* = 0.34, *p* = n.s.). This makes sense given that the attention control workshop was not designed to encourage participants to think of themselves as a collective or to build connections with one another.

Similar to the intervention group, changes in PrEP adoption among control arm participants had no effect on their friendship dynamics: PrEP adopters were no more or less likely to form friendships with other network members (*b* = 0.11, *p* = n.s.) nor were they more or less likely to form friendships with other PrEP adopters (*b* = 0.26, *p* = n.s.). Instead, older participants were more likely to form new Facebook friendships with other network members (*b* = 0.02, *p* < 0.01) and, like the intervention arm, new friendships were more likely to form among control arm participants who were similar in age (*b* = 0.78, *p* < 0.001). 

However, unlike intervention participants, friendships among control participants were not influenced by their HIV status. Namely, control participants who were living with HIV were no more or less likely than HIV negative participants to form friendships with other network members (*b* = −0.10, *p* = n.s.), and they were no more or less likely to sort based on HIV status similarities (*b* = −0.005, *p* = n.s.). Further, control participants were more likely to form new Facebook friendships if they had an existing offline relationship in the form of a study referral tie (*b* = 1.46, *p* < 0.001), unlike their intervention counterparts.

Finally, the structure of the network itself also influenced network dynamics among control participants. In line with results of the intervention model (Model 1), the formation of new friendship ties in the control network were more likely to ensure network closure (*b* = 0.58, *p* < 0.001) and were more likely to form on the basis of degree similarity (*b* = 0.06, *p* < 0.01). Further, the effect of network isolation was positively significant (*b* = 2.69, *p* < 0.001), indicating a positive tendency toward network isolation.

**Figure A1:**
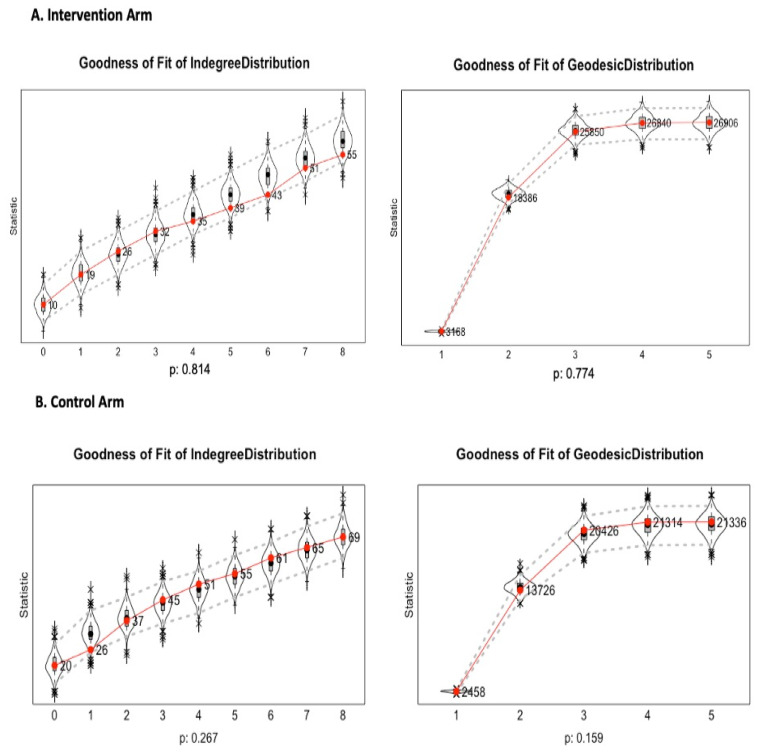
Goodness of fit statistics for the (**A**) intervention arm and (**B**) control arm models.

Modeling the PrEP adoption process among control participants yielded similar results to those from the intervention model. The rate of PrEP adoption among control participants was not influenced by their Facebook friendships, neither through their exposure to friends who were PrEP adopters (*b* = 0.41, *p* = n.s.) nor through their connections to influential PrEP adopters (*b* = −0.02, *p* = n.s.). Likewise, the rate of PrEP adoption was significantly predicted by the intrinsic effect of HIV status (*b* = −1.99, *p* < 0.05) and marginally influenced by PrEP adoption intentions (*b* = 1.12, *p* < 0.10). Goodness of fit results for both models are available in Appendix B (see Figure A1). 

## 4. Discussion

The purpose of this study was to evaluate the impact of a PrEP peer leader intervention on a large cohort of YBMSM peer leaders, with a specific interest in understanding whether and how the activation of study participants as peer leaders altered their online network and PrEP behavior dynamics. Our analysis showed that online tie formation among participants in both arms of the study increased during the first 12-months of the intervention and, for the intervention participants, this increase was partially attributed to their participation in the peer leadership training program. Specifically, candidate peer leaders who participated in the same small-group peer leader training workshop at the onset of the intervention were more likely to form new Facebook friendships with one another during their 12-month enrollment. Our findings also suggest that the increase in connectivity among peer leaders can also be attributed to characteristics of the peer leaders themselves, namely their HIV status, their PrEP intentions, and their age. Peer leaders who were living with HIV were more active in forming new friendships with other peer leaders and were more active in forming new friendships with one another. Further, HIV negative peer leaders who had greater PrEP intentions were also more likely to form new connections with other peer leaders during the study, and new friendships were more likely to emerge between peer leaders who were similar in age. 

Although our analysis shows that PrEP adoption did increase among both sets of participants during the first 12-months of the study, those increases were seemingly unrelated to their network dynamics. To rule out the possibility that our findings regarding the absence of social influence effects on PrEP adoption were the result of having too few PrEP adopters in either arm, we performed supplementary analysis (not shown here) of the PrEP adoption process in the unstratified sample using the same model specifications applied to the stratified samples. Results of this supplementary analysis (not shown here) reveal similar results: neither social influence term (total exposure or infection by degree) played a significant role in PrEP adoption. 

Our findings have implications for future peer leadership interventions and their role in community capacity-building. To begin, we are encouraged by the fact that the peer leadership training, a seminal component of the implementation of the intervention, played a significant role in encouraging the formation of new ties among newly activated peer leaders. Although we cannot say for sure that peer leaders perceived an increase in their individual and collective capacities as a result of their involvement in the training session, the fact that new online friendships were forged among members of the same training cohort after their training suggests that the relational momentum behind collective capacity was initiated. 

We interpret the social effect of the training to be related to three aspects of its implementation. First, the training curriculum itself was explicitly designed to empower peer leaders to see themselves as a collective. That the trainings were conducted in small group settings was intentional, as it encouraged a cohort mentality and helped create the sense of a shared experience. Second, a Facebook group was created as part of the study to serve as a PrEP information repository and communication channel for peer leaders during their enrollment in the study. Participants were informed about this group during their training session. Although participants were not required to participate in group conversations or connect with other group members, it is likely that having access to the Facebook group enabled these connections and communication exchanges to emerge voluntarily. Third, several study events were held for peer leaders during their 12-month enrollment that brought training cohorts together to celebrate their work and to exchange experiences engaging with peers about PrEP. These, too, provided additional opportunities for connection and potential capacity-building. For these reasons, it seems clear that organizing additional social opportunities for peer leaders, where they can connect and support one another, will help nurture their collective capacity as community health leaders. 

Another set of noteworthy findings pertain to the role that PrEP itself played in the formation of new friendships among PrEP peer leaders. Although 15 peer leaders adopted PrEP during their tenure in the peer leader role, their changes in behavior did not influence their friendship dynamics with other peer leaders. In other words, we did not observe PrEP adopting peer leaders playing a more active role in strengthening the social fabric among peer leaders. We take this as an indication that being an engaged peer leader is not necessarily contingent on taking PrEP. It has been surmised that drawing on community members who have personal experiences with the behavior of interest is an optimal strategy for selecting candidate peer leaders, as this may increase their enthusiasm and engagement in the study [59,60], as well as their self-efficacy [61]. However, findings from previous work and the current study suggest otherwise. In prior work, we learned that PrEP adopters were no more or less likely to recruit others into the study or to complete check-in calls with study staff [20], and in the current study we learned that this extends to their likelihood of connecting with other peer leaders. 

That said, controlling for peer leaders who had already adopted PrEP and who were living with HIV, we learned that peer leaders who had greater intentions to adopt PrEP at the start of the intervention were significantly more likely to form new relationships with other peer leaders. As such, it seems as though having greater interest in taking PrEP in the near future may be a critical motivation for being more engaged in the study and wanting to build community with other peer leaders, as these particular peer leaders could relate to the intended audience of the intervention (i.e., YBMSM who are good candidates for PrEP). With this in mind, it may be wise to recruit candidate peer leaders who demonstrate greater interest in taking PrEP themselves. Additionally, these findings also suggest that more effort could have been made to motivate PrEP-adopting peer leaders to take a more active role in strengthening the capacity of other peer leaders, for example by asking them to share their experiences being on PrEP with other peer leaders and to take on a leadership role within the peer leader cohort.

Also worth highlighting is the role that people living with HIV played in building community capacity among peer leaders. Despite not being eligible for PrEP themselves, we surmised that people living with HIV could be a powerful voice in bringing attention to a biomedical tool that can prevent the transmission of HIV and liberate status discordant couples. Our findings provide some preliminary evidence for this intuition: peer leaders living with HIV were more likely to contribute to the increased connectivity of the peer leader network and were more likely to do so as a cohort, as evidenced by the fact that they were more likely to form new ties with other participants living with HIV. That this dynamic was only evident in the peer leader network (as opposed to the control arm network) suggests that the intervention itself may have been a motivating factor. Pragmatically speaking, the tendency for PrEP peer leaders who are living with HIV to forge new friendships among themselves could be leveraged in the implementation of the intervention, for example by helping them coordinate their outreach efforts as a cohort.

Our results also point to a structural effect that has implications for community capacity-building. In both sub-samples, we found significant positive effects of network closure (i.e., the tendency to form friendships with the friends of your friends) on friendship formation. Given Facebook’s “People You May Know” Recommender, which suggests people you should connect with based on mutual friendships, it is unsurprising that network closure was a significant positive predictor of network change among study participants in both the intervention and control arms. In the context of a peer leadership intervention, network closure can have advantages and disadvantages. On one hand, network closure can generate bonding social capital [62] by nurturing trust, support, and solidarity among peer leaders. Research has shown that these social assets can be critical for capacity-building, specifically for creating AIDS-resilient communities [63]. Therefore, future research and implementation planning should be directed toward devising strategies to leverage the bonding capital that often emerges between peer leaders toward sustaining their coordinated outreach and engagement in the focal community. On the other hand, too much network closure, particularly if it occurs among peer leaders who are more trepidatious or less effective as peer leaders, could close these individuals off from new perspectives that could increase their confidence in the role. For this reason, supporting bonding social capital should not come at the cost of nurturing bridging capital, especially when it brings together peer leaders with different skill sets and different levels of confidence.

Finally, it is also worth discussing what we did not find. Namely, the increase in PrEP adoption between baseline and 12-month observation points in both intervention and control arms was seemingly unrelated to social influence processes as observed on Facebook. In many ways these null results are unsurprising given the nature of the network and the scope of the larger information environment in which the study took place. With respect to the network, peer influence on an individual’s PrEP decision making may have been more likely to occur in the context of more intimate physical world relationships that were unobserved, for example in the context of confidant relations or sexual partnerships. As we were unable to account for the potential influence of online peers who were not in the study, it is also possible that our Facebook network may have been missing some of its more influential actors and ties. 

This study should be interpreted in the context of several limitations. First, previous research has shown that offline and online relationships, particularly Facebook friendships, have a tendency to overlap [64,65]. However, the effort required to form online connections and, therefore, the meaning of those relationships make them notably different from offline relationships. Precisely how community leaders’ online relationships and the communication that occurs within them contribute to community capacity is an open question that requires more attention. Second, our singular focus on the network dynamics among peer leaders presents only a partial picture of the relational infrastructure from which community capacity is built. Although it was beyond the scope of this study, future research should be directed at understanding the effect of peer leader interventions such as PrEP Chicago on the formation of relationships between newly activated peer leaders and members of the larger community to which they belong (e.g., YBMSM peers, community leaders and organizations). Third, so that we could effectively compare how study participation differentially influenced the network and behavioral dynamics of intervention and control arm participants, we chose to treat participants in each arm as two mutually exclusive sub-groups. However, this forced us to remain agnostic to the fact that online friendships also existed across groups. Whether and how the intervention influenced the network and behavior dynamics between conditions is a question that needs further exploration and which has implications for our understanding of the intervention’s impact.

## 5. Conclusions

Despite these limitations, this is the first study to our knowledge that fully considers the effect of a peer leader intervention on the co-evolving relationships and behaviors of the peer leaders themselves and to unpack those dynamics in terms of their implications for community capacity-building. To these ends, we applied novel longitudinal social network models to determine the relationship between the online friendship networks of a cohort of PrEP peer leaders and their personal PrEP adoption behaviors, followed by a comparison analysis of the same dynamics among a cohort of control participants. Peer leaders actively shaped their online social environment by forming friendships with other members of their training cohort and who were similar in age and HIV status. Although PrEP adoption did not motivate the formation of new friendship ties, having greater PrEP intentions did. In comparison, online friendships among control participants were unaffected by their co-participation in a training session, their HIV status, PrEP adoption, and PrEP intentions. Although our findings are in part specific to the PrEP Chicago intervention, our goal was to articulate and apply a joint theoretical and analytic framework that help us see and evaluate peer leadership interventions and their social and behavioral effect on peer leaders as critical ingredients of longer-term capacity-building efforts in communities that are undergoing social and behavioral change. 

## Figures and Tables

**Table 1:** Description of the effects included in the network and adoption process sub-models.

Effect (Parameter Name)	Description
**Facebook Network Dynamics**	
Behavior Effects (PrEP adoption)	
Behavior of actor (egoPlusAltX)	Effect of the behavior (PrEP adoption) on friendship formation
Same behavior Facebook friend (sameX)	Preference to form friendships based on same behavior (both actors are PrEP adopters)
Study Effects	
Same training group assignment Facebook friend (sameX)	Preference to form friendships with participants assigned to the same training group
Additional Covariate Effects (age, HIV status, PrEP intentions)	
Actor covariate (egoPlusAltX)	Effect of the attribute on friendship formation
Same or similar covariate Facebook friend (sameX or simX)	Preference to form friendships with peers who share the same categorical or continuous trait
Dyadic identity (W)	Effect of having a referral relationship on friendship formation
Structural Effects	
Degree (density)	Tendency to form friendships
Network Closure (gwesp)	Preference to form friendships with the friends of current Facebook friends
Preferential Attachment (degPlus)	Preference to form friendships with highly connected network members
Degree Assortativity (outInAss)	Preference of high degree actors to form friendships with other high degree network members
Isolate (outIso)	Tendency for network isolates to remain isolated
**PrEP Adoption Process**	
Contagion Effects	
Total exposure (totExposure)	Total number of friends that are PrEP adopters
Infection by degree (infectDeg)	The infectiousness of highly connected PrEP adopters (influence determined by degree)
Intrinsic Effects (treatment assignment, age, HIV status, PrEP intentions)	
Actor covariate (RateX)	Effect of an actor attribute on the rate of PrEP adoption

**Table 2:** Characteristics of YBMSM study participants, stratified by intervention and control group assignment in Year 1.

	Intervention Arm (*n* = 174)		Control Arm (*n* = 166)	
	Wave 1	Wave 2	Wave 1	Wave 2
Binary Characteristics	N (%)	N (%)	N (%)	N (%)
PrEP Adoption	10 (5.8)	25 (14.4)	11 (6.6)	20 (12.0)
HIV positive	74 (42.5)	--	71 (42.8)	--
PrEP intentions (in next 6 months)				
Probably/definitely would not take PrEP	24 (14.7)	--	16 (10.4)	--
Might take PrEP	77 (47.2)	--	74 (48.1)	--
Probably/definitely would take PrEP	62 (38.1)	--	64 (41.6)	--
Continuous Characteristics	Mean (SD)	Mean (SD)	Mean (SD)	Mean (SD)
Age	26.1 (4.3)	--	25.3 (4.0)	--
Number of referrals	0.8 (0.8)	--	0.7 (0.8)	--
Number of training group co-members	4.7 (2.1)	--	3.8 (1.8)	--

**Table 3:** Structural properties of the intervention and control arm Facebook friendship networks at baseline and 12-months.

	Intervention Arm		Control Arm	
Characteristic	Baseline	12-Months	Baseline	12-Months
Mean (SD) of Facebook friendships	13.1 (10.8)	17.2 (13.6)	9.8 (9.2)	13.8 (12.3)
Network density	0.08	0.10	0.06	0.08
Edge count	1140	1497	813	1146
Transitivity	0.27	0.30	0.25	0.31
Centralization	0.16	0.23	0.18	0.23
	Period 1		Period 1	
Number of new Facebook friendship ties	555		533	
Number of stable Facebook friendship ties	942		613	
Number of dissolved Facebook friendship ties	198		200	
Jaccard Index ^a^	0.56		0.46	

^a^ The Jaccard index measures the amount of change between observed waves, and indicates whether the data collection points are not too far apart. Values greater than 0.3 are desired to meet assumptions that the network change process is gradual [36].

**Table 4:** Significance of parameter estimates of the Facebook network and PrEP adoption process sub-models.

	Intervention Arm		Control Arm	
Effect	b	(SE)	b	(SE)
**Facebook Network Dynamics Sub-model**				
Structural effects				
Basic rate parameter	6.31	(0.31)	7.68	(0.37)
Degree (density)	−3.10 ***	(0.31)	−2.97 ***	(0.33)
Network closure (gwesp)	0.26 *	(0.12)	0.58 ***	(0.13)
Preferential Attachment (degPlus)	0.005	(0.01)	−0.003	(0.01)
Degree Assortativity (outInAss)	0.06 **	(0.02)	0.06 **	(0.02)
Isolates (outIso)	1.96	(1.26)	2.69 ***	(0.68)
Behavior effects				
PrEP adoption actor	0.14	(0.30)	0.11	(0.32)
Same PrEP adoption Facebook friend	0.14	(0.36)	0.26	(0.39)
Study effects				
Same training group assignment Facebook friend	0.92 ***	(0.17)	0.34	(0.22)
Other actor covariate effects				
Age of actor	0.01	(0.01)	0.02 **	(0.007)
Similar age Facebook friend	1.30 ***	(0.21)	0.78 ***	(0.22)
HIV status actor	0.19 **	(0.06)	−0.10	(0.07)
Same HIV status Facebook friend	0.18 *	(0.08)	−0.005	(0.08)
PrEP intentions actor	0.13 **	(0.05)	0.07	(0.05)
Same PrEP intentions Facebook friend	−0.01	(0.14)	0.22	(0.14)
Dyadic covariate effects				
Study referral tie	0.67 ^†^	(0.37)	1.49 ***	(0.43)
**PrEP Adoption Process Sub-model**				
Contagion effects				
Rate of period 1	0.31	(0.35)	0.12	(0.13)
Total exposure	0.13	(1.89)	0.41	(2.71)
Infection by degree	−0.01	(0.06)	−0.02	(0.10)
Instrinsic effects				
HIV status actor	−1.34 *	(0.65)	−1.99 *	(0.98)
PrEP intentions actor	0.51	(0.42)	1.12 ^†^	(0.67)

Note: Convergence t-ratios < 0.07 and 0.05 and overall maximum convergence ratio = 0.17 and 0.20 for intervention and control arm models, respectively. ^†^ *p* < 0.10, two-tailed; * *p* < 0.05, two-tailed; ** *p* < 0.01, two-tailed; *** *p* < 0.001, two-tailed.

## Data Availability

The data presented in this study are available on request from the corresponding author. The data are not publicly available due to privacy and ethics requirements.

## Appendix A

### The Logic of Stochastic Actor-Based Models

SABMs and their variants are actor-oriented models, which means they conceive the co-evolution of networks and behaviors as happening at the hands of individual actors who make decisions to change their relationships or their behaviors in the name of optimizing their position in the network [37]. Changes between each wave of observed data are modeled using continuous-time Markov chains to determine the most likely series of unobserved micro-steps taken by actors when changing their network ties or their behaviors. As a continuous-time Markov chain process, the micro-changes to network ties and behaviors are assumed to occur sequentially. Further, the model assumes that actors can change a network tie or change their behavior (or make no change), and are assumed to react to changes made by other network actors [36].

Social and behavioral changes that occur between each wave of observed data are captured in two components of the model: the rate function captures the speed by which the dependent network or dependent behavior changes; and the evaluation function determines the “rules” that motivate these changes [36]. These rules are parameterized in the model as model effects, the estimates for which allow us to infer which rules were most likely to guide the unobserved micro-steps that led to larger observed changes to the dependent variables [36]. These effects can be purely structural, whereby individual changes are made in response to the presence or absence of ties around them, or attribute-based.

## Appendix B

### Model Goodness of Fit

To determine whether the selected models for the intervention and control arm samples provide a good fit to the observed network dynamics, we assess the fit of each model with respect to degree distribution and geodesic distribution. In RSiena, the goodness of fit (GOF) function operates by comparing the observed values at the end of a period with the simulated values for the end of a period. The differences are assessed by combining the auxiliary statistics using the Mahalanobis distance [66]. A model is considered an acceptable fit to a particular auxiliary statistic if the *p*-value of the Mahalanobis distance is larger than the conventional threshold of α = 0.05.
